# Invited Review: Maintain or Improve Piglet Gut Health around Weanling: The Fundamental Effects of Dietary Amino Acids

**DOI:** 10.3390/ani11041110

**Published:** 2021-04-13

**Authors:** Shengfa F. Liao

**Affiliations:** Department of Animal and Dairy Sciences, Mississippi State University, Mississippi State, MS 39762, USA; s.liao@msstate.edu; Tel.: +1-662-325-7318

**Keywords:** gut health, weanling pig, amino acid, gastrointestinal tract, feeding strategy, gut function

## Abstract

**Simple Summary:**

Gut health has significant implications for swine production. Gut morphology and luminal microbiota play determinant roles for its health maintenance. Amino acids, a group of nutrients for pigs, are not only obligatory for maintaining gut tissue integrity, but also for supporting the growth of luminal microbiota. This review summarized the up-to-date knowledge concerning the effects of dietary amino acids on the gut health of weanling pigs. Studies have shown that different amino acids have similar but also some different effects on gut health, but how to take advantages of all these effects for field application is not clear. The interactions between the effects of non-nutrient feed additives and the fundamental effects of amino acids warrant further investigation. Considering the global push to ban the antibiotics usage for swine production, a primary effort at present may be made to explore the specific and then the concert effects of amino acids on the profile and functions of gut microbiota in young pigs.

**Abstract:**

Gut health has significant implications for swine nutrient utilization and overall health. The basic gut morphology and its luminal microbiota play determinant roles for maintaining gut health and functions. Amino acids (AA), a group of essential nutrients for pigs, are not only obligatory for maintaining gut mucosal mass and integrity, but also for supporting the growth of luminal microbiota. This review summarized the up-to-date knowledge concerning the effects of dietary AA supplementation on the gut health of weanling piglets. For instance, threonine, arginine, glutamine, methionine and cysteine are beneficial to gut mucosal immunity and barrier function. Glutamine, arginine, threonine, methionine and cysteine can also assist with relieving the post-weaning stress of young piglets by improving gut immunological functions, antioxidant capacity, and/or anti-inflammatory ability. Glutamine, glutamate, glycine and cysteine can assist to reconstruct the gut structure after its damage and reverse its dysfunction. Furthermore, methionine, lysine, threonine, and glutamate play key roles in affecting bacteria growth in the lumen. Overall, the previous studies with different AA showed both similar and different effects on the gut health, but how to take advantages of all these effects for field application is not clear. It is uncertain whether these AA effects are synergetic or antagonistic. The interactions between the effects of non-nutrient feed additives and the fundamental effects of AA warrant further investigation. Considering the global push to minimize the antibiotics and ZnO usage in swine production, a primary effort at present may be made to explore the specific effects of individual AA, and then the concert effects of multiple AA, on the profile and functions of gut microbiota in young pigs.

## 1. Introduction

Indisputably, a healthy gastrointestinal tract, also known as the gut, is very critical for the overall nutrient metabolism, physical activities, body wellbeing, and production efficiency of pigs at every stage of life. It is especially important at the post-weanling stage when piglets experience diarrhea in various degrees which can last for 1 or 2 weeks. The diarrhea or gut disturbances shortly after weaning normally cause enormous economic losses to the swine industry, and indeed, during the weanling period, piglets achieve probably less than 50% of their growth potential even under an optimal husbandry condition [1]. The increased litter size (12~15 or more piglets/litter), reduced birth weight (on average) and the early weaning practice (from 6~8 weeks of age in the past to 3~4 weeks of age or even younger nowadays) in modern swine operations have led the pork industry to even more challenges [2]. This is why there is a wide range of interests in the field of swine science and production in developing both management and feeding strategies to stimulate gut development and enhance gut health in weanling piglets [3,4,5]. The ultimate goal of these strategies is to improve the productivity of the newly weaned piglets, while minimizing or banning the use of antibiotics, zinc oxide (ZnO) and rather expensive feed ingredients such as milk products [1,5].

For the feeding strategies with dietary intervention, a substantial number of studies have been conducted evaluating the impact of nutrients, feed ingredients, and non-nutrient feed additives (those additives that are not nutritionally or physiologically required by the pig) on various aspects of gut development, health, and functions in young piglets, and excellent review papers on the topic were published by de Lange et al. [1], Lallès et al. [3], Xiong et al. [5], Heo et al. [6], and Muns and Magowan [7], to name a few. Amongst those feed additives, antibiotics and ZnO are widely used in the industry to alleviate these weaning-associated gut health problems and to improve pig growth performance. However, the recent bans on the in-feed use of antibiotics and pharmacological doses of zinc in the European Union and many other countries have or will have negative impact on the control of weaning diarrhea [6,8,9]. Here, it is not an intention of this article to further review the vast amount of information in the literature pertaining to the topic of in-feed additives; instead, the focus of this article is to highlight the fundamental effects of dietary amino acids (AA), without using feed additives, on stimulating gut development, health and functions in piglets in the neighborhood of weanling practice.

Besides the text review of the literature in the following six sections, Appendix A Table A1 provides an overview of the research during the last two decades concerning the effects of some important AA on the health and functions of gastrointestinal tract in weanling pigs. It needs to be pointed out that although the emphasis of this article is on the weanling piglets, some theoretical concepts and/or practical effects may be relevant to other categories of pigs as well.

## 2. Amino Acid Effects on Gut Morphology

Gut morphology, or the anatomical and histological structure of the gastrointestinal tract, is definitely the physical foundation for the good number of biochemical and physiological functions performed by the gut, such as feed digestion and nutrient absorption [10,11]. The most measured parameters indicating gut morphological integrity and health status are villus height (VH), crypt depth (CD), and VH:CD ratio (VCR). The villi are finger-like projections into the intestinal lumen while the crypts are invaginations between the villi [11]. Increased VH may result in a greater absorptive capability for available nutrients, whereas reduced CD indicates a decrease in metabolic cost of epithelium turnover [12]. So a higher CD value (meaning a deeper crypt) suggests a faster epithelium turnover for villus renewal as needed in response to the inflammation from pathogens or chemical toxins. The study of Min et al. [13], however, explained that deeper CD indicates increased enterocyte proliferation, increased villus surface area, increased mucin secretion (because goblet cells are mainly present in the crypts) and, therefore, better nutrient absorption. In general, however, a notion commonly accepted is that a reduction in VH and an increase in CD, or a reduced VCR, in small intestine are associated with villous atrophy that will, in turn, lead to a reduction in the specific activities of brush-border enzymes, such as lactase and sucrase [14].

Glutamine (Gln) participates in many key metabolic processes, such as protein and nucleic acid bio-syntheses, gluconeogenesis, and inter-organ nitrogen transfer [15]. Dietary Gln supplementation can decrease the susceptibility of enterocytes and lymphatic cells to apoptosis [16]. Glutamate (Glu), which can be produced from Gln by glutaminase in small intestine, is a specific precursor for the intestinal synthesis of glutathione (GSH), arginine (Arg) and proline (Pro) [15]. Although whether or not Glu can effectively substitute Gln in the diet under any conditions remains unknown, it has been shown that the provision of supplemental Gln or Glu to suckling and weanling piglets improved their whole-body health and growth, which was related to the improvement in gut morphology and health [17,18]. Cabrera et al. [19] reported that supplementation of creep feed and nursery diets with Gln and/or AminoGut (a commercial feed supplement containing 10% Gln and 10% Glu) in the first three weeks post-weaning improved feed conversion in piglets, possibly due to the improvement in gut health. The 1% Gln group had the greatest VH while the 0.88% AminoGut group had the deepest CD among the dietary treatments [19]. Positive effects of dietary supplementation of Gln, including alanyl-Gln or glycyl-Gln, on the small intestinal mucosal morphology (including the increased VH, reduced CD, and increased VCR) in weanling pigs have been reported in several previous studies [20,21,22]. Rezaei et al. [23] reported that dietary supplementation of 1% to 4% Glu (in monosodium form) increased the jejunal VH in post-weaning pigs. Lin et al. [24] reported that the addition of Glu (at 2%) improved the small intestinal architecture, the jejunal mucosal mass and barrier function of weaning piglets, which is beneficial for the improvement of nutrient digestion and absorption. Having said that, there are also inconsistent results in the literature. Domeneghini et al. [25] reported that dietary supplementation of 0.5% Gln above NRC [26] requirement resulted in an increase in VH, CD, and a decrease in VCR in weanling gilts. He et al. [18] failed to observe any beneficial effects of Gln and Glu combination on gut morphology in weaned piglets although a tendency of improvement was shown. This discrepancy may be due to the starting age of the piglets (7 days post-weaning), which might have passed the critical stage [18].

Arginine is a conditionally essential AA for pigs in all production phases [27], and the Arg content is remarkably deficient in sow milk for suckling piglets [28] and it is also relatively low in typical corn and soybean meal–based diets (without the addition of plasma or fish meal) for weanling pigs [29]. The Arg biosynthesis by weanling pigs is also insufficient, resulting from inadequate intake of other AA (e.g., Gln, Glu, and Pro) and low activity of mitochondrial N-acetylglutamate synthase [29,30]. Wu et al. [31] reported that supplementing 0.6% Arg to a corn and soybean meal–based diet increased the VH in duodenum, jejunum, and ileum, as well as the CD in jejunum and ileum of the 21- to 28-day-old weaned pigs, although the VCR was not altered. Yang et al. [32] reported that supplementation of 0.8% Arg in pre-weaning diet (milk replacer) improved the VH in duodenum, jejunum, and ileum, villous area in duodenum and jejunum, and CD in ileum of the piglets. And a positive carry-over effect on gut development and the subsequent whole-body growth performance were also observed during the immediate post-weaning period [32].

Sulfur-containing AA, such as methionine (Met) and cysteine (Cys), are also beneficial for the maintenance of gut morphology [33]. Luminal microbes are responsible for the extensive catabolism of dietary Met in the gut [15]. Chen et al. [34] reported a higher VH in jejunum and a lower CD in duodenum, as well as an increased VCR in all three sections of the small intestine in the Met-supplemented (vs. Met-deficient) post-weaning piglets. A study on nursery/weanling pigs also showed that adding a liquid Met analogue (an acid form) in drinking water can improve gut morphology by increasing the VH in duodenum, jejunum and ileum, and the VCR in jejunum and ileum [35]. Shen et al. [36] reported that dietary Met for post-weanling piglets enhanced the duodenum VH which was associated with the reduced oxidative stress and improved GSH production in the mucosa cells. Cys is extensively utilized by animal gut [15]. Bauchart-Thevret et al. [37] concluded that the gut of weanling pigs utilizes 25% of the dietary Cys intake, and that the synthesis of mucosal epithelial bioactive compounds, such as GSH and mucin, are the major non-oxidative fate of Cys metabolism.

Threonine (Thr), with a high utilization rate by the gut, is well involved in intestinal maintenance and functionality [38,39,40]. It was reported that Thr is a major component of mucins (40% of the mucus glycoproteins) in the gut [13]. Dietary Thr supply is critical for gut morphological maintenance and development because Thr plays a key role in mucin synthesis and barrier integrity [13,15,41]. Ren et al. [42] and Koo et al. [43] both reported that dietary supplementation of Thr above the NRC [23] requirement enhanced intestinal morphology as reflected in the improved VH, CD, and VCR in weaned pigs. The density of goblet cells in the jejunal villi and crypts was also increased upon dietary Thr supplementation [43]. Either deficiency or excess of dietary Thr has adverse effects on the synthesis of intestinal mucosal proteins such as mucins in young pigs [44].

Finally, dietary supplementation of tryptophan (Trp) can improve VCR in the small intestine, but it may depress voluntary feed intake and growth of the piglets around weanling [45,46]. Dietary supplementation of aspartate (Asp) at 0.5 to 1.0% can also enhance intestinal integrity and energy status in weanling piglets after lipopolysaccharide (LPS) challenge [9]. In terms of Pro, Wang et al. [47] reported that an oral administration of Pro (25 mg/kg BW) improved mucosal proliferation, intestinal morphology, as well as tight junction and potassium channel protein expression in early-weaned piglets.

## 3. Amino Acid Effects on Gut Luminal Microbiota

Gut luminal microbiota, a collection of microorganisms that form a complex micro-ecosystem, have been recognized to have broad biological effects not only on the gut health but also on the whole-body health and growth performance of pigs [48,49]. It was shown that dietary AA have significant effects on the metabolism and composition of gut microbiota that primarily consist of bacteria [50,51]. The major metabolites from gut bacteria fermentation are short chain fatty acids (a.k.a. volatile fatty acids) which play a key role in maintaining gut health by lowering luminal pH and regulating microbial population, especially by stimulating the growth of beneficial bacteria [52]. Extensive fermentation of undigested feed components by the bacteria in large intestine such as cecum is responsible for detoxification of harmful substances and prevention of the colonization of pathogenic bacteria [53].

Threonine is mostly metabolized in the gut for incorporation into functional proteins including mucins and γ-globulins [54]. Evidence is growing that dietary Thr supplementation (at 0.3%) beneficially modifies the gut microbiota composition in poultry [52,55] and rats [56]. Dong et al. [52] reported that dietary Thr supplementation to a low crude protein diet for laying hens recovered the bacteria diversity (caused by the low protein diet) and increased the abundance of potential beneficial bacteria. One of the explanations for this effect might be the up-regulation of mucin gene expression, because mucins cannot be digested in small intestine and thereby will reach ceca, acting as a substrate for saccharolytic bacteria. In nursery pigs, Koo et al. [43] reported that the supplementation of Thr 15% above the NRC [27] requirement (on the standardized ileal digestible basis) seemed to modify intestinal microbial fermentation in an interactive manner with diet composition. Thr supplementation in a simple diet (contained soybean meal as a protein source) decreased the intestinal NH_3_ content but increased the intestinal volatile fatty acid content when compared with a complex diet (contained various sources of animal protein) [43].

As is known, the extensive catabolism of dietary lysine (Lys) in the gut is taken care of by luminal bacteria rather than the enterocytes and, therefore, it is postulated that dietary Lys restriction can dramatically affect gut microbiota [15]. Yin et al. [57] firstly reported that Lys restriction enhanced the intestinal richness and evenness of microbial community. Moreover, using a bioinformatics software package, Yin et al. [57] also predicted that the altered intestinal microbiota caused by Lys restriction might influence AA metabolism, membrane transport, endocrine system, carbohydrate metabolism, as well as cellular signaling, replication and repair.

A study on nursery pigs showed that an addition of a liquid Met analogue (a free acid form) in drinking water tended to decrease the gastrointestinal pH and the concentrations of acetic acid in cecum. However, the total number of lactic acid bacteria and *E. coli* in cecum was not affected [35]. Thus, Kaewtapee et al. [35] concluded that the liquid Met analogue might enhance nutrient digestion and absorption (due to the increased VH) and, subsequently, the growth performance of pigs.

In addition, Liang et al. [58] reported that dietary supplementation of Trp (0.2 to 0.4%) altered the structural and functional composition of the intestinal microbiota in weanling piglets. The abundances of *Prevotella*, *Roseburia*, and *Succinivibrio* genera were enriched, but those of opportunistic pathogens, such as *Clostridium sensu stricto* and *Clostridium XI*, were decreased [58]. Feng et al. [59] reported that Glu (a monosodium form) can markedly increase the diversity of intestinal microbial community in growing pigs by promoting the colonization of *Faecalibacterium prausnitzii* and *Roseburia*.

## 4. Amino Acid Effects on Gut Immunological Functions

Gut is the largest immune organ in the body with its mucosal defense mechanisms being subdivided into two arms—the innate immunity and acquired/adaptive immunity—which are integrated and work together to protect the gut and, in turn, the whole body from diseases [49,60]. In a broad sense, the innate immune system is composed of anatomic, physiologic, phagocytic, and inflammatory barriers, while the adaptive immune system is composed of specialized, systemic cells and processes to eliminate specific pathogens. The aforementioned barriers, although oftentimes overlooked, however, provide the first line of defense eliminating 99.9% of all infections [61,62]. In addition, the gut mucosal surface forms an intricate collaboration with the intestinal lumen. The diverse milieu of dietary antigenic components, as well as the various populations of microbes, in the lumen have facilitated the need for an evolving and sophisticated mucosal immune system [60]. The mucosal immune system contains more than a trillion lymphocytes and has a greater concentration of antibodies than any other tissue has in the body [60]. It protects the gut against harmful pathogens but also tolerizes or induces tolerance to commensal microorganisms and dietary antigens [62].

Studies have shown that some AA are more crucial in the maintenance of gut immunological function than others [63,64]. Glutamine supplementation can stimulate both innate and adaptative arms of immunity, as shown by the increased densities of macrophages and intra-epithelial lymphocytes [3]. Wu et al. [65] reported in a mouse model that Gln supplementation may enhance intestinal secretory IgA production in the gut through regulation of intestinal microbiota and/or the T cell-dependent and -independent pathways.

In terms of Arg, numerous studies have identified its important role in the gut immunity of humans and animals [15,63]. As a central intestinal metabolite, Arg functions as a regulatory molecule limiting intestinal alterations and maintaining gut immunological functions, in addition to its role as a constituent of protein synthesis [66,67,68]. Corl et al. [69] reported that early in rotavirus enteritis, Arg had a beneficial effect on the intestinal barrier system of piglets by reducing trans-epithelial permeability via a mammalian target of rapamycin/p70S6k-independent mechanism.

Threonine is an essential component of mucus (approximately 35~40% of mucins or the mucus glycoproteins) in the gut [13,70]. Threonine supply thus is crucial for maintaining gut immunological functions by participating in mucin synthesis and maintaining gut barrier integrity [15,41,70]. Moreover, Thr has been reported to be an AA with the highest concentration in the γ-globulins of rabbits, horses and humans. In young pigs, the humoral antibody production and serum specific IgG concentrations were all increased in response to the increased intake of true ileal digestible Thr [15]. Zhang et al. [71] reported that supplementation of Thr (at a level of 0.2%) improved the intestinal mucin synthesis and immune function of the intrauterine growth-retarded weanling piglets. Chen et al. [55] reported that the jejunal and/or ileal IgG, IgM, and SIgA contents, as well as the goblet cell density, were increased by the dietary supplementation of Thr (at the level of 0.1 or 0.3%). All these results indicate that Thr is very important for the protection of gut mucosal barrier and for gut immunological functions.

A sufficient supply of sulfur-containing AA, Met and Cys, from diet or from tissue protein breakdown, is necessary for the synthesis of a myriad of proteins and peptides involved in the normal functions of the immune systems of pigs [72]. Methionine serves as a methyl donor for several important processes, such as DNA methylation and polyamine synthesis [73], which are important for enhancing immune cell proliferation during an immune challenge. During the immune system stimulation (ISS), utilization of Cys for the production of compounds involved in immune response, such as taurine and GSH, is largely increased [74]. Taurine is particularly abundant in leucocytes, a group of immune cells found throughout the body [75]. Rakhshandeh et al. [76] reported that the immune system stimulation by injection of LPS reduced the ratio of whole-body nitrogen to sulfur balance, indicating that the sulfur-containing AA are preferentially preserved for the production of non-protein compounds, such as GSH, to enhance the whole-body immune status. These results imply that more Met and Cys are needed during the immune challenge state in pigs.

## 5. The Anti-Oxidative Functions of Amino Acids

The animal gut has quite complex antioxidant systems that are regulated at the level of vitagenes operating network [77]. In piglets, weanling stress is the main cause of the gut barrier function loss and the gut inflammation and dysfunction, which are associated with the increased free radical generation, decreased antioxidant defenses, and reduced digestive enzyme activities [77]. Free radicals, especially the reactive oxygen species (ROS), can damage cellular macromolecules, such as proteins, lipids, and DNA, and these damages can induce cellular oxidative stress and impair the integrity of gut mucosal epithelium, which, in turn, can lead to serious problems to animal health [78,79]. Nutritional measures to assist or enhance animal endogenous antioxidant systems is especially critical during the pig weanling practice [80].

All AA are susceptible to oxidation, although their susceptibilities vary considerably [81,82]. Several AA can execute their antioxidant functions through GSH that is a major cellular antioxidant, functioning to detoxify the intestinal oxidative stress and injuries that are involved in the microbe-induced inflammation [37]. Methionine and Cys are considered as endogenous antioxidants because Met and Cys are most susceptible to the oxidation by ROS [83,84]. The antioxidative ability of Met can protect many proteins from oxidative damage [85]. Dietary Met for nursery pigs enhanced the duodenum morphology in association with reduced oxidative stress and improved GSH production in mucosa cells [36,72]. Some complex, but coordinated, responses of antioxidant systems were observed in different tissues when growing pigs faced an insufficient dietary Met supply [86]. Cysteine along with Glu and glycine (Gly) is an important precursor of GSH. Taurine is another sulfur-containing AA produced from Cys, and it is also a cellular antioxidant, as well as a cell membrane stabilizer [73].

Amongst various AA, Glu and Gln are the major oxidative fuels for small intestinal mucosa. The dominant role of Glu as a fuel may have therapeutic potential for improving the function of the infant gut, because the rate of the gut epithelial cell turnover is high [87]. Yin et al. [88] reported that Glu alleviated the diquat-induced oxidative stress via enhancing the superoxide dismutase (SOD), total antioxidant capacity (T-AOC), and nitric oxide (NO) levels and inhibiting lipid oxidation subsequent with malondialdeyhde (MDA) generation. Similar to Glu, Gln also provides fuel for the rapidly dividing cells, particularly, the lymphocytes and enterocytes, as well as other intestinal epithelial cells [15,89]. Kim [89] recounted that dietary Gln supplementation has beneficial effects in reducing the symptoms of inflammatory disorders and protecting the gut against the damages from the oxidative stress. Gln is easily converted to Glu by glutaminase in the small intestine, so Glu could be a preferable fuel to Gln for enterocytes when the activity of glutaminase was low [15]. A recent study in growing pigs, however, showed that dietary supplementation of monosodium L-Glu had detrimental effects on several physiological and inflammatory parameters measured in the proximal intestine, while exerting some beneficial effects on the distal intestine [90].

Tryptophan has antioxidative effects against oxidative stress, and Trp metabolites, such as melatonin, 3-hydroxykynurine, and 3-hydroxyanthranilic acid, are also antioxidants [91,92]. In weanling pigs, the results of Lv et al. [93] showed that a diquat-induced oxidative stress decreased the Trp concentration in serum and the 5-hydroxytryptamine concentration in the hypothalamus, and increased kynurenine (a large neutral AA) and MDA in serum. However, whether Trp administration will be beneficial enough to pigs under oxidative stress still need further research. Shen et al. [94,95] reported that dietary Trp supplementation reduced the MDA content in plasma and hypothalamus, which indicated the antioxidative effects of Trp against lipid peroxidation. In terms of Arg, Zheng et al. [96] reported that dietary supplementation can attenuate the diquat-induced oxidative stress through the enhancement of antioxidant capacity in weanling piglets.

## 6. The Anti-Inflammatory Effects of Amino Acids

Inflammation is a physiological protective response involving immune cells, blood vessels, and molecular mediators, and it is one of the first responses of the immune systems to infection, harmful irritation, or oxidative stress by the mechanism of innate immunity [62,97]. The intestinal immune responses could lead to inflammatory responses and secretion of antibodies [49,98]. The intestinal inflammation is associated with increased gut permeability that may lead to translocation of toxins, allergens, viruses, or even bacteria [49]. Measurement of pro-inflammatory cytokines can provide some information as to the degree of local mucosal inflammation [98,99].

Studies have shown that some AA can alleviate intestinal inflammation. Using a mouse model, Chau et al. [100] reported that dietary Arg supplementation reduced the expression of ileal transcript mRNA encoding interleukin-4 (IL-4), a key mediator of intestinal mastocytosis and macromolecular permeability. The data suggested that increasing bioavailable Arg ameliorates intestinal inflammation and pathology. It is likely that the altered activities of Arg-catabolizing enzymes, such as arginases and NO synthase (NOS), contribute to ameliorating the allergic inflammation. It can be postulated that the activity of arginase enhances epithelial barrier function through conversion of Arg into ornithine. Recent studies showed that NOS activity can dampen inflammation through regulation of the myeloid and lymphoid cell activation [68]. Nitric oxide produced by inducible NOS in inflammatory monocytes and dendritic cells can regulate inflammatory cytokine production, cell differentiation, and survival [68]. Modulating the arginase- and NOS-mediated pathways through regulation of Arg or its precursor citrulline via oral supplementation can be an efficient and practical strategy to dampen intestinal inflammation and pathology and regulate the mucosal immunohomeostasis [68].

Ample evidence also demonstrated that Gly has efficacy as an anti-inflammatory and cytoprotective agent [101]. While the mechanism responsible for its protective effects is unclear, it is likely to be multi-factorial involving direct effects on target cells, inhibition of Gly-gated chloride channels, and/or inhibition of inflammatory cell activation. Some studies indicated that Gly has a protective effect in mesenteric ischemia/reperfusion injury through the inhibition of apoptosis [102], while others have shown that Gly protection against intestinal ischemia/reperfusion injury is reached by a mechanism that is consistent with Gly uptake [103]. Although it seemed that dietary Thr supplementation did not normalize the gut inflammation induced by feeding a soybean meal based simple diet (without animal proteins) to weanling pigs [43], Chen et al. [104] reported that Thr supplementation (at 3.0 g/kg diet) could attenuate LPS-induced inflammatory responses in young broilers, as indicated by the reversed LPS-induced increase in the concentrations of pro-inflammatory cytokine in serum, spleen, and intestinal mucosa.

## 7. Detoxification and Gut Dysfunction Reverse by Amino Acids

Intestinal barrier dysfunction caused by natural toxins or microbial toxins can open a “door” for invasion of exogenous pathogens or pathogenic antigens. Previous studies have shown that AA could alleviate the adverse effects of toxins and gut barrier dysfunction. Several studies demonstrated that dietary supplementation with Glu restores mucous circulation and AA metabolism, as well as prevents the enterocyte apoptosis [105,106]. Through a study with fifteen young growing pigs, Duan et al. [106] concluded that Glu may be useful as a nutritional regulation factor to alleviate the adverse effects of mycotoxins on gut structure (histology or morphology) and barrier functions, and whole-body growth performance, because dietary Glu supplementation counteracted partially the impairments induced by the mycotoxins from mold-contaminated feed.

Glutamine is a unique nutrient for enterocytes, capable of dual signaling and augmenting the effects of growth factors that govern cellular proliferation and reconstruction after damage [107]. Souba et al. [108] suggested that in humans Gln is an important AA for maintenance of gut structure, metabolism, and function, especially during critical illness when gut mucosal barrier is compromised. Kessel et al. [109] reported that an enteral feeding of Gln suppressed the injury to the mucous membrane of the small intestine caused by LPS endotoxemia in rat. In mice, dietary Gln supplementation can block ethanol-induced gut permeability and protect colonic epithelial tight junctions and adherent junctions [110,111]. A recent study conducted by Jiang et al. [112] using an endoplasmic reticulum stressed intestinal porcine epithelial cell (IPEC-J2) model induced by tunicamycin suggested that Gln can protect the cell from the stress-caused apoptosis via the inositol requiring enzyme 1 (IRE1)-X-box binding protein 1 (XBP1) axis. As is known, the IRE1-XBP1 axis is crucial to the maintenance of the gut endoplasmic reticulum homeostasis. Xue et al. [12] reported that dietary Gln supplementation improved the architecture of jejunum and ileum during the necrotic enteritis outbreak and recovery, and consequently favors the gut functions in broilers. In short, dietary Gln supplementation can maintain gut barrier function and reverse the gut barrier dysfunction, such as that induced by alcohol [89,110,111].

Glycine is not only an essential substrate for synthesizing several important biomolecules, such as glucose and GSH, but also is utilized in biochemical detoxification via conjugation of endogenous or xenobiotic toxins [113,114]. A study with a rat model suggested that local Gly perfusion diminished the ischemia-reperfusion injury in small intestinal mucosa, as indicated by the increased mucosal protein content, increased mucosal DNA content, and maintenance of mucosal glutaminase activity, during either the pre-ischemia phase or the pre-reperfusion phase [103].

Cysteine can modulate local cytokine gene expression, suppress pro-inflammatory and chemotactic gene expression, and promote the expression of pro-apoptotic pathways, in addition to its known immunological and antioxidant effects, suggesting that dietary Cys supplementation may support the recovery of gut mucosal homeostasis [115]. Lysine also serves as a partial antagonist of gut serotonin 5-HT4 receptors to reduce stress-related diarrhea as well as anxiety, and may modulate gut motor function [116]. Another study conducted by Yin et al. [117] on growing pigs showed that supplementing 1% Arg to a mold-contaminated feed ameliorated the intestinal abnormalities caused by mycotoxin, exerting a protective role against pig mycotoxicosis.

## 8. Conclusions Remarks

Feeding crystalline AA to pigs has been a commercial approach to raise pigs for optimal muscle growth. However, in recent years, plenty of knowledge pertaining to the roles of AA on gut health has provided ample scientific basis for nutritionists to reconsider the current AA requirements of pigs. As reviewed above, several AA are especially beneficial for the maintenance or improvement of gut health in weanling pigs. While some AA provide fuel for the growth and proliferation of the intestinal epithelial cells, others offer nutrients to luminal microbiota for maintaining its diverse composition and functions. Moreover, the types and levels of different AA can differently or similarly (i.e., overlapped) affect gut morphology and functions. Thr, Arg, Gln, Met and Cys are beneficial to protecting gut barrier function and maintaining gut mucosal immunity. Gln, Arg, Thr, Met and Cys could assist with relieving the post-weaning stress of young piglets by improving the immunological functions, anti-inflammatory ability, and/or antioxidant capacity. Gln, Glu, Gly, and Cys can assist to reconstruct the gut structure after its damage and reverse its dysfunction under disease conditions. Furthermore, Met, Lys, Thr, and Glu play important roles in affecting the growth of different bacteria in gut lumen.

From this review of the literature, it can be seen that nearly all the reported studies with individual AA supplementation above the NRC [26,27] recommendations showed beneficial effects on the intestinal health of weanling piglets, but how to comprehensively take advantages of all these effects for field application is not clear. It is uncertain whether these effects from different AA are synergetic, simple additive, or antagonistic. Currently. numerous non-nutrient feed additives are widely used in weanling practice to improve piglet gut health, although their beneficial effects are not evident in many cases [1,118]. The interactions between the added effects of those feed additives (including dietary fiber) and the fundamental effects of various AA also warrant further investigation. Considering the global push to ban the usage of in-feed antibiotics or the medicinal doses of ZnO as growth promoters for swine production, a primary effort at the present time may be made to explore the specific effects of individual AA as well as the concert effects of multiple AA on the profile and functions of gut microbiota in young pigs.

## Appendix A

**Table A1:** An overview of the research during the last two decades concerning the effects of dietary amino acids on gut health and functions in weanling pigs ^1^.

Amino Acid(s)	Dietary Concentrations ^2^	Major Effects on the Gut Health Parameters Observed	Reference
Arginine	0.6 vs. 0.0%	Increased the small intestinal growth, the villus height (VH), the crypt depth (CD), and the goblet cell counts in the mucosa	Wu et al. [31]
Arginine	1.0 vs. 0.0%	Increased the epithelial VH and the mucosal vascular endothelial growth factor level of the small intestine; Reduced the CD in the duodenum and jejunum	Yao et al. [119]
Arginine	1.0, 0.5, vs. 0.0%	Protecting and enhancing intestinal mucosal immune barrier function, and maintaining intestinal integrity after *E. coli* lipopolysaccharide (LPS) challenge	Zhu et al. [120]
Arginine	0.8, 0.4, vs. 0.0%	Increased (linearly and quadratically) the VH, villus area, and CD; Increased (linearly and quadratically) the mucosal protein content	Yang et al. [32]
Arginine	1.6, 0.8, vs. 0.0%	Decreased the CD and suppressed the inflammatory cytokine expression in the jejunum	Zheng et al. [121]
Glutamine	2.0 vs. 0.0%	Mitigated villus atrophy and morphology disruption of the gut after *Escherichia coli* challenge	Yi et al. [20]
Glutamine	0.5 vs. 0.0%	increased both VH and CD, decreased the VH to CD ratio (VCR); Increased in mitotic mucosal cells (M), decreased in apoptotic mucosal cells (A), thus decreasing the A:M index; The percentages of mucosal macrophages were greater	Domeneghini et al. [25]
Glutamine	0.5 vs. 0.0%	Increased the VH and CD and decreased the VCR; Gut barrier function may be improved	Domeneghini et al. [16]
Glutamine	4.4 vs. 0.0%	Reducing the mucosal cytokine response; Improving the intestinal barrier function	Ewaschuk et al. [122]
Glycyl-glutamine	0.15 vs. 0.00%	Mediated the adverse effects of *E. coli* LPS on gut integrity; the proinflammatory response may be limited	Jiang et al. [21]
Alanyl-glutamine	0.45, 0.30, 0.15, vs. 0.00%	Increased the VH and VCR in duodenum and jejunum; the digestive-absorption function may be enhanced via those digestive enzymes and nutrient transporters analyzed	Zou et al. [22]
Glutamate	1.0 vs. 0.0%	Increased the VH and mucosal thickness in the jejunum; Having favorable effects on gut epithelium cell proliferation	Wu et al. [123]
Glutamate	2.0 vs. 0.0%	Improved the intestinal integrity; influenced the expression of amino acid receptors and transporters in the jejunal mucosa	Lin et al. [24]
Glutamate	2.0 vs. 0.0%	Alleviated the diquat-induced oxidative stress via enhancing the superoxide dismutase, total antioxidant capacity, and nitric oxide levels and inhibiting lipid oxidation subsequent with malondialdeyhde generation.	Yin et al. [88]
Monosodium glutamate	4.0, 2.0, 1.0, 0.5, vs. 0.0%	Increased jejunal VH, DNA content, and antioxidative capacity; reduced the incidence of diarrhea	Rezaei et al. [23]
Glutamine + Glutmate	(1.0 + 0.0), (0.9 + 0.1), (0.8 + 0.2), vs. (0.0 + 0.0)	The combinational effects of glutamine and glutamate could not achieve that of glutamine alone	He et al. [18]
Glutamine; AminoGut ^3^	1.00; (0.88 to 0.66), vs. 0.00; 0.00%	Increased the jejunal VH by glutamine; increased the jejunal CD by AminoGut (glutamine + glutamate)	Cabrera et al. [19]
Methionine	0.12 vs. 0.00%	Increased the VH in jejunum, decreased the CD in duodenum, and increased the VCR in all three sections; Increased the abundance of occludin and decreased the abundance of active caspase-3 in the jejunum	Chen et al. [34]
Methionine	0.12 vs. 0.00%	Improved intestinal integrity and oxidative status	Su et al. [124]
Methionine	0.145 vs. 0.000%	Enhanced the duodenum morphology in association with reducing oxidative stress; Improved glutathione production in the mucosa cells	Shen et al. [36]
Methionine hydroxy analogue-free acid	0.10, 0.05, vs. 0.00%	Tended to decrease pH in the stomach, duodenum, jejunum, colon and rectum; The 0.10% group increased the VH in duodenum, jejunum and ileum, and the VCR in jejunum and ileum	Kaewtapee et al. [35]
Cysteine	0.61 vs. 0.00%	Increased the synthesis of mucosal epithelial proteins, such as glutathione and mucin	Bauchart-Thevret et al. [37]
N-acetyl cysteine	500 vs. 0 mg/kg	Possessing a constructive regulation on the changes of gut redox status and microbiota in response to weaning stress	Xu et al. [125]
Taurine	0.1 vs. 0.0%	Decreased the stimulation of immune response to lipopolysaccharide; Improved intestinal epithelial barrier function	Tang et al. [126]
Sulfur amino acids ^4^	0.96, 0.85, 0.74, 0.63, vs. 0.53%	Improved intestinal digestive and absorptive functions via affecting the mucosal antioxidant systems in a dose-dependent manner	Zong et al. [33]
Tryptophan	0.5 vs. 0.0%	Increased the VH and VCR but unaffected transport of macro molecules (indicating the gut permeability)	Koopmans et al. [45]
Tryptophan	0.4, 0.2, vs. 0.0%	Altered intestinal microbial composition and diversity; Improved intestinal mucosal barrier function	Liang et al. [58]
Tryptophan	0.75, 0.15, vs. 0.00%	Negatively affected intestinal morphology (increased CD, decreased VCR) and decreased the mRNA expression of some tight junction proteins	Tossou et al. [127]
Threonine	3.2, 2.2, 1.2, 0.5, vs. 0.0 g/kg	Increased the humoral antibody production and serum specific IgG concentrations	Wang et al. [128]
Threonine	11.1, 7.5, vs. 3.7 g/kg	Improved the intestinal morphology and mucosa immune function; had beneficial effects in maintaining jejunal morphology integrity and repairing villous damage caused by *E. coli* challenge	Ren et al. [42]
Threonine	0.2 vs. 0.0%	Improved the intestinal mucin synthesis and immune function; attenuated ileal inflammatory responses, of the intrauterine growth-retarded weanling piglets	Zhang et al. [71]
Threonine	0.12 vs. 0.00%	Seemed to have greater benefits with a simple diet (contained soybean meal as a protein source) than with a complex diet (contained animal protein sources) in intestinal morphology, production of gut microbial metabolites, and inflammatory status in the jejunum	Koo et al. [43]
Branched-chain amino acids ^5^	Leu (1.38 vs. 1.26%), Ile (0.80 vs. 0.60%), Val (1.01 vs. 0.74%)	Enhanced intestinal development (increased VH or reduced CD), and intestinal expression of several amino acid and peptide transporters	Zhang et al. [129]
Aspartate	1.0, 0.5, vs. 0.0%	Attenuated LPS-induced intestinal damage indicated by greater VH and VCR as well as higher RNA/DNA and protein/DNA ratios; Improved intestinal function indicated by increased mucosal disaccharidase activities; Improved intestinal energy status indicated by increased ATP, ADP and total adenine nucleotide contents, adenylate energy charge and decreased AMP/ATP ratio	Pi et al. [9]
Lysine ^6^	1.60, 1.23, vs. 0.86%	Lysine restriction inhibited intestinal lysine transport, and enhanced the richness and evenness of the microbiota composition and diversity	Yin et al. [57]
Amino acid blend (AAB) ^7^	1.00% AAB vs. 0.99% alanine	Improved the intestinal morphology, barrier function, and antioxidative capacity; Reduced the diarrhea incidence; Enhanced the intestinal expression of the heat shock protein-70 gene	Yi et al. [130]

^1^ The terms of weaning pigs, weanling pigs, weaned pigs, weaner pigs, postweaning pigs, nursery pigs, and young piglets after weaning used by different researchers for this topic of study are all classified subjectively as weanling pigs in this table. ^2^ Unless specified, each value indicates a supplemental concentration on the top of the basal diet which already met the dietary requirement for the said amino acid. The last concentration on the list for each study is, in nearly all the studies, for the basal diet (a control group). ^3^ AminoGut (Ajinomoto do Brazil, São Paulo, Brazil) contained 10% glutamine and 10% glutamate. ^4^ Sulfur amino acids (SAA) contain methionine and cysteine. These concentrations (calculated) corresponded to 130, 115, 100, 85, and 70%, respectively, of the SAA:Lysine ratio that had been recommended by NRC [27]. ^5^ Branched-chain amino acids, including leucine (Leu), isoleucine (Ile) and valine (Val), were added to a low protein (17.9%) diet to meet the recommendations. The concentrations of other essential amino acids were at the same levels between the two diets. ^6^ The lysine concentrations (calculated) corresponded to 130, 100 and 70%, respectively, of the dietary lysine requirement recommended by NRC [26] or [27]. ^7^ The AAB included glutamate:glutamine:glycine:arginine:N-acetylcysteine at a ratio of 5:2:2:1:0.5 on a weight basis.
